# En Bloc Transplantation of Horseshoe Kidney from Deceased Donor: An Unusual Transplantation Utilizing Kidneys with Congenital Fusion Abnormality

**DOI:** 10.1155/2021/2286831

**Published:** 2021-08-11

**Authors:** Bodhisatwa Sengupta, Iftikhar Khan, Akram Saiaghi, Ethar A. Gaw, Mansour Tawfeeq, Mohammed S. AlQahtani, Mahmoud Obeid

**Affiliations:** Department of Transplant Surgery, King Fahad Specialist Hospital, Dammam, Saudi Arabia

## Abstract

Transplanting horseshoe kidneys is challenging and has higher complication rates due to the unusual anatomy of the vascular and urinary collecting systems. Most centers avoid using these kidneys for transplantation. However, if chosen carefully, these organs can be used successfully to reduce organ shortage. In this paper, we will describe the technique of procurement of horseshoe kidneys from cadaveric donors, back table preparation, and its successful implantation in a recipient. With good planning and skillful surgical techniques, horseshoe kidneys can be successfully transplanted in suitable recipients. If properly selected, these kidneys can be used to reduce the organ scarcity and diminish waitlist morbidity and mortality.

## 1. Introduction

Horseshoe kidney is a congenital anomaly occurring due to abnormal fusion of the kidneys in fetal life. They can be considered selectively to address organ shortage for transplantation, especially in regions of known organ scarcity. The unusual anatomy, tenuous vascular supply particularly to the lower pole of kidney, anomalies in the urinary collecting system and the large size of the allograft result in an increased incidence of complications. This makes en bloc transplantation of horseshoe kidney a challenging undertaking requiring meticulous planning and skillful surgery. In this case report, we will share the experience of an en bloc horseshoe kidney transplantation, the various challenges we encountered, and how we overcame them.

## 2. Case Presentation

### 2.1. Donor

**Figure 1:**
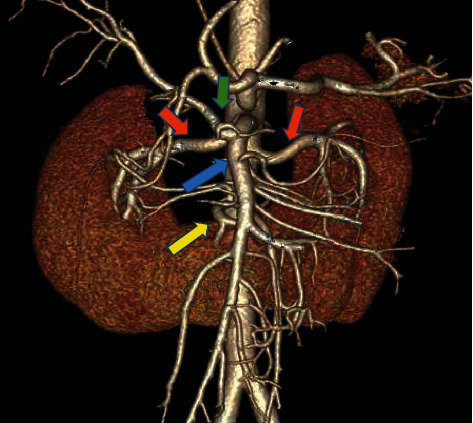
CT angiographic reconstruction of the vascular anatomy of the horseshoe kidney. Yellow arrow: the lower polar artery supplying the lower poles of both kidneys and the isthmus. Red arrows: the right and left main renal arteries. Blue arrow: superior mesenteric artery (SMA) originating from the aorta in very close proximity to the right renal artery. Green arrow: replaced right hepatic artery arising from the SMA.

**Figure 2:**
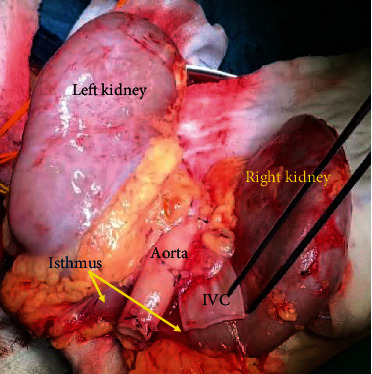
The posterior view of the horse kidney after procurement and completion of back table preparation, ready to be transplanted.

The donor was a young person who was hemodynamically stable and was declared brain dead following intracranial hemorrhage. Past medical history was not significant for infections or malignancies. Computerized tomography (CT) using intravenous contrast was done for possible splitting of the liver and revealed horseshoe kidneys (Figure 1). The right kidney was 12.5 cm long, the left kidney measured 13.7 cm, and both were joined at their lower pole. The arterial supply of each kidney was through a single main renal artery. The isthmus and the lower poles shared blood supply from a single lower polar renal artery arising adjacent to the inferior mesenteric artery. The right renal artery (RRA) originated at the level of the superior mesenteric artery (SMA) separated from it by 3 mm. The right hepatic artery (RHA) was completely replaced, originating from the SMA. The isthmus of the horseshoe kidney was fleshy, 5 cm wide, and about 2.5 cm thick (Figure 2). The cold perfusion cannula was placed in the right common iliac artery to ensure good flow in the abnormal lower polar branch supplying the lower poles and isthmus. The horseshoe kidneys were procured en bloc with the intact aorta and inferior vena cava (IVC). The aorta was transected proximally at the level of SMA and distally at the aortic bifurcation.

### 2.2. Back Table Preparation

The en bloc graft weighed 450 gm and was 13.7 cm long and 20 cm wide (Figure 2). The proximal end of the aorta could not be closed primarily by oversewing with sutures or by using staplers because of the close proximity of the orifice of RRA to the cut margin. Instead, we sutured an aortic patch circumferentially to the transected edge to close the proximal end. The superior end of the IVC was closed primarily using running nonabsorbable suture. The inferior ends of the aorta and the IVC were kept open and were prepared for anastomosis. The inferior mesenteric artery and the gonadal veins were ligated, and the fat around the kidneys was trimmed.

### 2.3. Recipient

**Figure 3:**
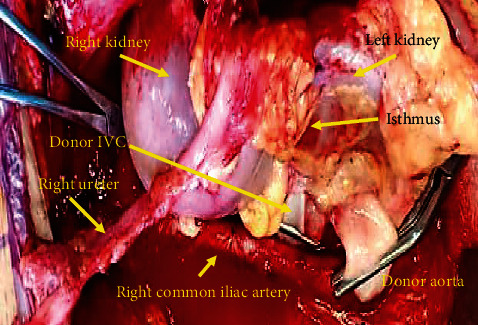
Picture taken before reperfusion of the horseshoe kidney, immediately after completion of anastomosis between inferior end of donor aorta and IVC to the common iliac artery and vein, respectively.

**Figure 4:**
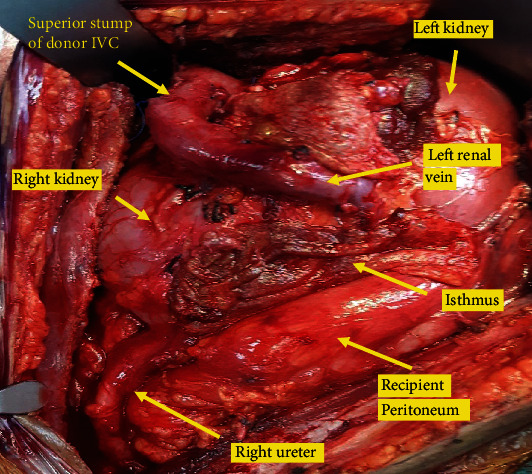
Anatomy of horseshoe kidney allograft in the retroperitoneal space after reperfusion.

**Figure 5:**
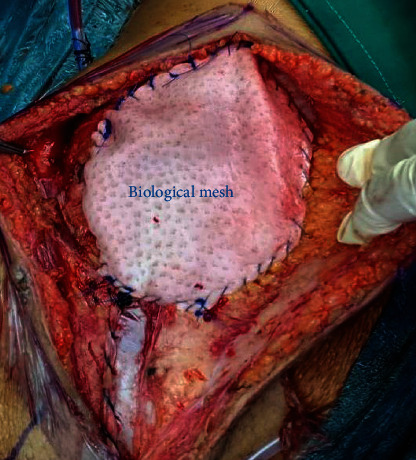
Partial closure of wound using a biological mesh in order to reduce tension in the wound and prevent increased intra-abdominal pressure secondary to the large allograft size.

The recipient was petite having a height of 150 cm. The patient was on peritoneal dialysis (PD) for end-stage renal disease secondary to Alport's disease. Informed consent for the procedure was obtained after explaining the potential complications, risks, and benefits of kidney transplantation with emphasis on those associated with the use of horseshoe kidneys. Under general anesthesia, a right-sided hockey stick incision was made starting from the pubic tubercle. Considering the size of the en bloc horseshoe kidney, the incision was made longer than usual so as to extend up to few centimeters below the right costal margin. The PD catheter was in the right lower abdomen in close proximity of the incision and was removed early during surgery. The peritoneum was medialized extensively to create a large retroperitoneal space to accommodate the allograft. The inferior part of the IVC and the aorta were exposed. The entire length of common iliac artery and vein was dissected circumferentially, and vascular control was established. The en bloc kidneys were placed within the retroperitoneal space, and the inferior ends of the aorta and IVC were trimmed to appropriate lengths and then anastomosed end to side to the common iliac artery and vein respectively using 6.0 polypropylene sutures (Figure 3). The kidneys perfused very well and started making urine intraoperatively (Figure 4). Cold ischemia time was 21 hours and warm ischemia time was 40 minutes. The two ureters were next anastomosed separately to the bladder over 6F 14 cm double J stents using 5.0 polydioxanone sutures. Biological mesh was used for abdomen closure to avoid high intra-abdominal pressure (Figure 5). Postoperatively, the patient required low dose vasopressor support and was kept overnight in the intensive care unit. During this period, muscle relaxants were used and she was kept intubated and ventilated. Intra-abdominal pressure was monitored. Postoperative urine output ranged between 5 and 8 ml/kg/hour. The creatinine level decreased from preoperative 1263 mmol/L to normal range of 93 mmol/L in 2 days. Patient was subsequently transferred to the floor and was discharged on the 6^th^ day after surgery. Intraoperative and follow-up Doppler ultrasound showed excellent perfusion of the kidneys including the isthmus. Immune suppression included induction with Thymoglobulin and maintenance with Tacrolimus and Mycophenolate orally as per our center protocol. The two ureteric stents were removed by cystoscopy 4 weeks after the surgery. The patient is now more than 42 weeks since surgery, and she had an uneventful recovery. Her latest creatinine is 55 mmol/L.

## 3. Discussion

Horseshoe kidneys are possibly the commonest congenital fusion abnormality of the renal system occurring in up to 0.25% (1 in 400) of the population [1]. Embryologically, this develops from abnormal migration of nephrogenic cells occurring as early as the 4^th^ to 6^th^ week of gestation [1, 2]. This results in a fleshy isthmus joining the lower poles of the two kidneys and assuming the shape of a horseshoe. In 5%, the fusion can occur in the upper pole. In 15%, the fleshy isthmus can be replaced by fibrous tissue [2]. Since this fusion occurs before the medial rotation of the kidneys, the ureters can be found to lie anteriorly [3]. Normal vascular anatomy comprising of a single renal artery supplying each kidney is seen in just 30% cases [4]. Majority of those diagnosed to have horseshoe kidney remain asymptomatic. Symptoms arising from complications due to hydronephrosis, infection, and stones occur in up to 40% of patients [5].

The number of published cases are few due to the rarity of this congenital defect and the difficulty of the surgery [1, 2, 6, 7]. The first transplantation of horseshoe kidney was performed by Politano in 1963 and Nelson was the first to describe the technical aspects in 1975 [2, 8].

Transplantation of horseshoe kidney is challenging due to the complexity of the vascular supply and anomalous collecting system [6]. These kidneys can be transplanted either en bloc or can be transplanted separately to two different recipients after isthmusectomy provided they have suitable anatomy of the vascular and collecting systems [7]. We opted to transplant en bloc as isthmusectomy could have resulted in ischemic injury to the lower pole of at least one kidney due to its shared vascularity. There have been reports of the development of ischemia and urinary leak in the transplanted kidneys after isthmusectomy [2, 3, 9]. As a result, division of the horseshoe kidney can result in rendering one of the kidneys nonusable due to these injuries. In deceased donors, the transection of the isthmus, if considered, should be done in the back table [10]. Few cases of living donor heminephrectomy from a donor with horseshoe kidney after transecting the isthmus have been described [7].

The cannula for cold perfusion was inserted in the right common iliac artery to avoid injury to the lower polar artery. Tan et al. [10] recommended cannulation of the common iliac arteries to avoid injury to the lower polar vessels and ensure proper cold perfusion through them. The transection of the proximal end of the aorta was challenging because the replaced RHA required an intact SMA and the ostium of the RRA was 3 mm away from it. Primary closure of the cut end of the proximal aorta would result in compromising the RRA. We used a patch of the aorta to close the proximal stump of the aorta in order to prevent any narrowing of the right renal artery ostium.

Considering the complexity of the transplant and size of the allograft, we wanted to select a recipient who had a spacious abdomen, had good cardiac output, was not coagulopathic, and could maintain satisfactory blood pressure to ensure good perfusion to this large-sized graft. The first recipient on the list was declined due to significant preoperative hypotension. A second recipient was called and resulted in the prolongation of the cold ischemia time. This patient was 150 cm tall, weighed 40 kg. Considering the slight build, we anticipated a small abdominal domain and planned to make a longer incision, extensive mobilization, closure of abdomen with mesh, and postoperative elective ventilation with muscle relaxation in the ICU. Postoperative US was done to confirm adequate vascularity of the graft. During the ICU stay, the intra-abdominal pressure was monitored to rule out the development of intra-abdominal and renal allograft compartment syndrome.

A preoperative CT scan with contrast was done for our donor to assess the vascularity of the liver and it incidentally revealed the horseshoe kidneys. Although CTA in this case helped us planning in advance, CTA for deceased donors are not practiced universally [11]. We feel that a preoperative CTA may provide the retrieval surgeon relevant information in case of anomalous anatomy and he can make appropriate preparations leading to successful transplantation of such organs. Over-the-phone communication with a senior colleague can be of big help and every effort should be made to utilize these kidneys for a successful outcome in the recipient.

## 4. Conclusion

We present this case report to emphasize that horseshoe kidneys are used to increase the donor pool. We also share our experience on how we overcame the challenges from the complicated anatomy, complex vascularity, and the mechanical disadvantage of a large size en bloc horseshoe kidney.

## Data Availability

The laboratory and radiological data are available with hospital electronic medical records.
